# Pleomorphic Adenoma of the Breast: A Rare Benign Lesion Masquerading as a Metaplastic Breast Carcinoma on Core-Needle Biopsy

**DOI:** 10.7759/cureus.38827

**Published:** 2023-05-10

**Authors:** Muhammad Ahmad, Anam Naumaan, Carlos Munoz Zuluaga, Esther Yoon

**Affiliations:** 1 Pathology and Laboratory Medicine, MD Anderson Cancer Center, Houston, USA; 2 Pathology and Laboratory Medicine, Weill Cornell Medicine/New York-Presbyterian Hospital, New York City, USA; 3 Pathology and Laboratory Medicine, Cleveland Clinic Florida, Weston, USA

**Keywords:** pathology, salivary gland, breast, metaplastic carcinoma, pleomorphic adenoma

## Abstract

We report a rare case of pleomorphic adenoma (benign mixed tumor) of the breast in a 66-year-old female. A 5.5 cm hypoechoic mass with lobulated margins was noted on ultrasound. A biopsy showed an atypical cartilaginous lesion, leading to a subsequent segmental mastectomy, which was initially interpreted as metaplastic breast carcinoma. On the second review at our tertiary care center, a diagnosis of a pleomorphic adenoma was favored due to the circumscription and the benign epithelial component. Due to unfamiliarity with this entity, this neoplasm has occasionally been misdiagnosed clinically and even been overcalled on core needle biopsies. Careful clinical, radiological, and pathological correlation is required to avoid unnecessarily aggressive surgery, and a differential diagnosis of pleomorphic adenoma must be included in cases of well-demarcated breast masses showing myxoid or cartilaginous changes on core-needle biopsy.

## Introduction

Pleomorphic adenoma (benign mixed tumor) of the female breast is a rare, benign neoplasm that morphologically resembles its counterparts in the salivary gland and the skin. There are only scattered case reports of these lesions in the breast [1]. Some authors dispute the existence of this tumor as a distinctive entity and have regarded it as a form of intraductal papilloma with extensive cartilaginous metaplasia [2]. The neoplasm is characterized by a proliferation of epithelial cells, myoepithelial cells, and mesenchymal stroma. Correct diagnosis is often challenging by clinical examination and radiological studies, and any atypia seen on core biopsies can be overcalled and misdiagnosed as malignant, particularly metaplastic breast carcinoma, leading to unnecessarily aggressive treatment for the patient. Here, we report a case of pleomorphic adenoma of the breast, which was previously diagnosed as a metaplastic carcinoma at an outside institution. We highlight diagnostic pitfalls of the entity and summarize current literature to gain insight and helpful clues when encountering similar lesions in the breast.

## Case presentation

**Figure 1:**
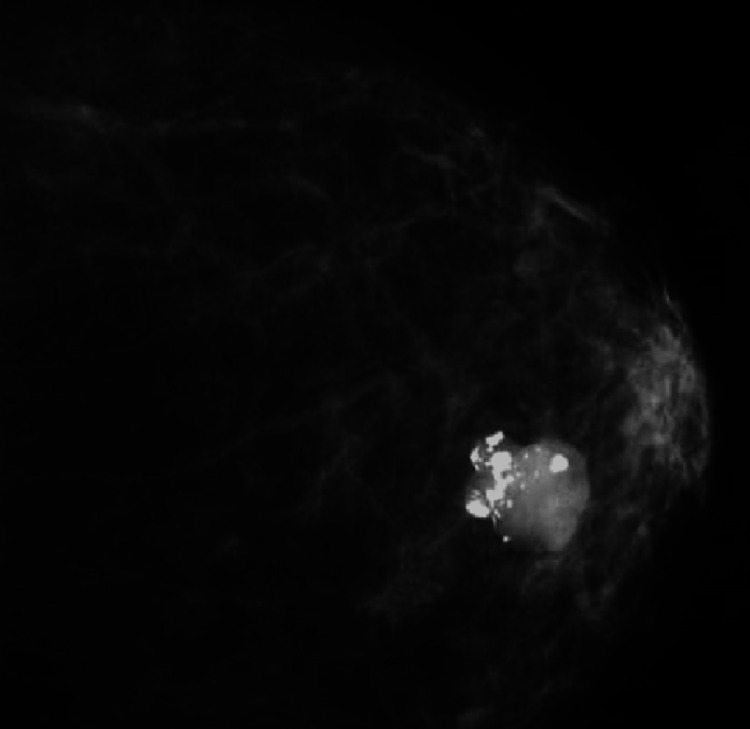
Ultrasound showing a hypoechoic mass in the left breast with lobulated margins and coarse calcifications

A 66-year-old woman presented with a 5.5 cm mass in the inferior inner quadrant of the left breast. Clinical examination was unremarkable for skin or nipple changes. Ultrasound demonstrated a hypoechoic mass with lobulated margins and coarse calcifications (Figure 1), and a biopsy was recommended. A biopsy performed and interpreted at an outside institution showed an atypical cartilaginous lesion, prompting a subsequent segmental mastectomy, which was interpreted as metaplastic breast carcinoma with chondroid and osseous differentiation. Three weeks after her surgery, the patient came to our tertiary cancer center for subsequent treatment, and the slides for both the initial core biopsy and the subsequent resection specimens were reviewed by the breast pathologists.

Materials and methods

Specimen processing was performed at the referring institution and followed standard protocol. The biopsy and resection specimens were fixed in formalin and embedded in paraffin, and 3-5 μm-thick tissue sections were used for staining with hematoxylin and eosin (H&E) and immunohistochemistry. The referring institution performed estrogen receptor (ER), progesterone receptor (PR), human epidermal growth factor receptor 2 (HER2)/neu, p63, Ki-67, pancytokeratin, CK5/6, and high-molecular-weight cytokeratin immunohistochemistry on both biopsy and resection specimens.

Results

**Figure 2:**
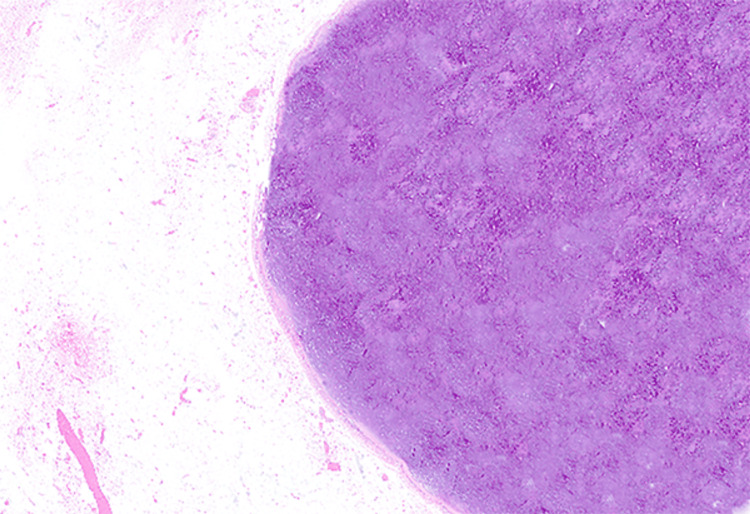
Low-power view showing a well-circumscribed neoplasm composed predominantly of cartilaginous components (X 40, H&E). H&E: hematoxylin and eosin stain

**Figure 3:**
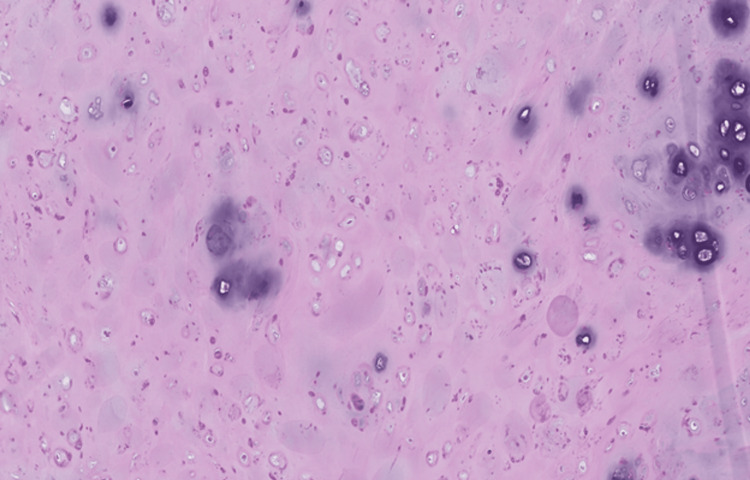
Medium-power view showing that the cartilaginous component exhibits focal minimal to mild cytologic atypia (X 100, H&E). H&E: hematoxylin and eosin stain

**Figure 4:**
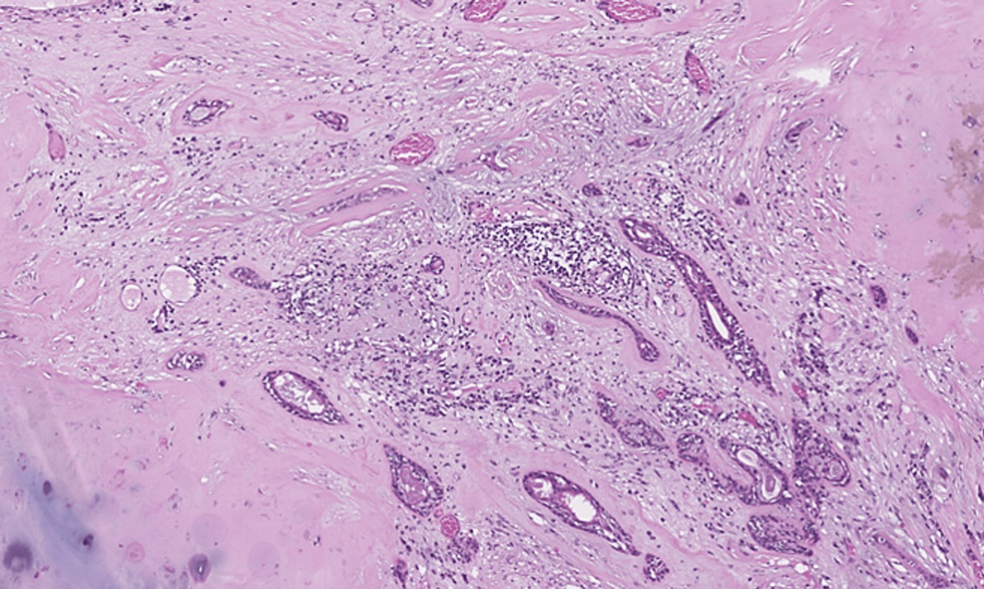
Medium-power view showing the ductal epithelial cells with myoepithelial cells and well-formed tubules (X 100, H&E). H&E: hematoxylin and eosin stain

Per gross description, the specimen showed a well-circumscribed mass measuring 5.5 cm in the greatest dimension. Microscopic review of the H&E-stained slides showed a well-circumscribed biphasic neoplasm composed predominantly of a cartilaginous component with a focal bland, glandular epithelial component (Figure 2). The cartilaginous component shows focal minimal to mild cytologic atypia (Figure 3). The ductal epithelial cells were noted to have surrounding myoepithelial cells forming predominantly tubules (Figure 4). The mild atypia seen in the chondroid component in the surgical specimen was similar to that in the patient's core-needle biopsy specimen.

Immunohistochemical studies showed that the neoplastic epithelial cells showed no nuclear expression for ER (<1% cells) and PR (<1% cells), and no membranous staining for HER2/neu (0). Ki-67 stain showed strong (3+) nuclear staining in 3% of cells while p63 stain showed moderate (2+) nuclear staining in 32% of tumor cells. The bland epithelial component showed positivity for pancytokeratin, CK5/6, and high-molecular-weight cytokeratin, whereas no expression for these markers was noted in the cartilaginous component.

Based on the morphologic and immunohistochemical findings, the differential diagnosis included a pleomorphic adenoma with focal and mildly atypical cartilaginous component, or a cartilaginous lesion of undetermined behavior. Due to the challenging nature of the case, slides were reviewed by multiple breast pathologists and head and neck pathologists. Overall, due to the well-circumscribed borders, the benign epithelial component, low Ki-67 proliferation index, and only mild atypia in the cartilaginous component, a diagnosis of pleomorphic adenoma with focally atypical cartilaginous component was favored and conservative management with close follow-up was recommended.

## Discussion

Pleomorphic adenoma is a benign mixed tumor commonly occurring in the salivary gland, and rarely originating in the breast. The tumor consists of epithelial, myoepithelial, and stromal components, which characteristically consist of bone and/or cartilage [3]. Differential diagnoses in the breast include fibroadenoma, phyllodes tumor, metaplastic carcinoma, and mucinous carcinoma. Pleomorphic adenomas in the core-needle biopsy could be misdiagnosed as a primary sarcoma or metaplastic carcinoma of the breast due to the abundance of metaplastic stroma. Therefore, this tumor can rarely present similar to breast cancer. Hence, 30-50% of patients who are initially misdiagnosed as having a malignant neoplasm on biopsy undergo unnecessarily aggressive surgery such as total, radical, or modified mastectomy with or without lymph node biopsy.

The first case of breast pleomorphic adenoma was described in French by Paul Lecéne in 1906 [4]. In English medical literature, it was initially described by Smith et al. in 1969 [5]. Occurrence of this neoplasm in the breast is exceedingly rare, and only case reports and small case series have been described in the literature. Our review of the literature shows that fewer than 80 cases have been published in the literature including the largest case series published by Ballance et al. in 1990 with 35 cases (Table 1).

**Table 1:** Reported cases of pleomorphic adenoma of breast in the medical literature

Case	Author	Age/Sex	Size (cm)	Location	Stroma	Treatment
1	Lecene et al.(1906) [4]	25/F	N/A	Right breast		Excisional biopsy
2	Lecene et al.(1906) [4]	54/F	N/A			Mastectomy
3	Nadal et al. (1910) [6]	44/F	N/A		Myxoid and chondroid	Mastectomy
4,5	D’Allaines et al. (1928) [7]	19/F N/A	N/A			Excisional biopsy, N/A
6	Gioia et al. (1930) [8]	42/M	4	Right breast	Myxoid and chondroid	Excisional biopsy
7	Nabert et al. (1968) [9]	38/F	N/A	Right breast	Myxoid and chondroid	Mastectomy
8	Poluektov et al. (1968) [10]	63/F	1	Right breast	Myxoid	Mastectomy
9-17	Smith and Taylor (1969) [5]	23-77/F	0.8-4.5		Chondroid x 9, osteoid x 6	Excision x 7 Mastectomy x 2
18	Paikova et al. (1972) [11]	39/F	4			Mastectomy
19	Kermarec et al. (1973) [12]	68/F	3	Right breast, subareolar	Myxoid and chondroid	Excisional biopsy
20	Williams et al. (1975) [13]	72/F	17	Right breast	Myxoid, chondroid and osteoid	Mastectomy
21	Jakimowicz and Gratama (1977) [14]	57/F		Right breast, subareolar	Chondroid	Excisional biopsy
22	Sheth et al. (1978) [15]	74/F	1.5	Right breast, subareolar	Myxoid, chondroid and osteoid	Excision
23	Medina and Uehlinger (1980) [16]	78/F	N/A	Left breast		Mastectomy
24-26	Makek and von Hochstetter (1980) [17]	35-78/ 2 F; 1 M	1.5-4	Right breast x 2, Left breast x 1	Myxoid x 2, chondroid x 2 and osteoid x 1	Excision x 2, Mastectomy x 1
27	McClure et al. (1982) [18]	46/F	2.0	Right breast, subareolar	Myxoid	Excision
28	Van der Walt and Rohlova (1982) [19]	67/F	2.5	Left breast, subareolar	Myxoid, chondroid and osteoid	Excision
29	Spagnolo and Shilkin (1983) [20]	46/F	1.5	Right breast, subareolar	Myxoid, chondroid and osteoid	Excision
30	Zafrani et al. (1985) [21]	66/F	2.0	Left breast, subareolar		Mastectomy
31	Segen et al. (1986) [22]	75/F	1.0	Left breast, subareolar	Myxoid and chondroid	Excision
32	Willen et al. (1986) [23]	76/F	2.5 and 1.7	Right breast, subareolar	Myxoid and chondroid	Mastectomy
33	Cuadros et al. (1987) [24]	65/F	2	Right breast, subareolar	Myxoid and chondroid	Excision
34	Soreide et al. (1988) [25]	61/F	1.8	Left breast, subareolar	Myxoid and chondroid	Excision
35	Ballance et al. (1990) [1]	77/F	2.0	Right breast, subareolar	Myxoid, chondroid and osteoid	Excision
36-41	Moran et al. (1990) [26]	37-85/F	1.0-4.0	Left breast x 2, right breast x 3		Excision x 5, Mastectomy x 1
42-43	Chen (1990) [27]	58, 75/F	0.7-1.3	Left breast, subareolar	Myxoid x 2, and chondroid x 2	Excision x 5, Mastectomy x 1
44	Nevado et al. (1991) [28]	84/F	0.8	Left breast, subareolar	Myxoid and chondroid	Excision
45-54	Diaz et al. (1991) [29]	50-68/ 9 F, 1 M	0.6-5.0		Myxoid x 10, chondroid x 6, and osteoid x 4	Excision x 8, Mastectomy x 2
55	Simha et al. (1992) [30]	65/F	2.5	Right breast	Myxoid and chondroid	Excision
56	Agnantis et al. (1992) [31]	62/F	3.2	Left breast, subareolar	Myxoid, chondroid and osteoid	Mastectomy
57	Narita and Matsuda (1995) [32]	70/F	1.0	Right breast, subareolar	Myxoid, chondroid and osteoid	Excision
58	Mochinaga et al. (1997) [33]	74/F	3.0	Left breast, subareolar	Myxoid and chondroid	Excision
59	Parham and Evans (1997) [34]	76/F	6.0	Left breast	Myxoid	Excision
60	Fiks (1999) [35]	43/F	1.2	Right breast	Myxoid, chondroid and osteoid	Excision
61	Reid-Nicholson et al. (2003) [36]	59/F	0.9	Right breast	Myxoid	Excision
62	Kumar et al (2004) [37]	47/F		Left breast	Myxoid	Excision
63	Sato et al. (2005) [38]	55/F	0.8	Right breast, subareolar	Myxoid and chondroid	Excision
64	De Dominicis et al. (2005) [39]	59/F	2.0	Left breast, subareolar	Myxoid and chondroid	Excision
65	Mizukami et al. (2008) [40]	76/F	1.5	Right breast	Myxoid and chondroid	Excision
66	Leekha et al. (2013) [2]	60/F		Left breast	Myxoid and chondroid	Excision
67	Khamechian et al. (2014) [41]	61/F	2.2	Right breast, retroareolar	Myxoid, chondroid and osteoid	Excision
68	Kelten et al. (2015) [42]	57/F	1.5	Left breast	Myxoid and chondroid	Excision
69	Kelten et al. (2015) [42]	78/F	1.5	Right breast	Myxoid and chondroid	Excision
70	Ginter et al. (2015) [43]	42/F	1.4	Left breast	Myxoid and chondroid	Excision
71	Rakha et al. (2015) [44]	F	1.6	Right breast, subareolar	Myxoid, chondroid and osteoid	Excision
72	Takahashi (2018) [3]	45/F	1.5	Left breast	Chondroid and osteoid	Excision
73	Moore et al. (2021) [45]	54/F	1.1	Left breast	Myxoid	Excision
74	Elaiwy et al. (2021) [46]	38/M	9.5	Left breast	Myxoid and chondroid	Excision
75	Elaiwy et al. (2021) [46]	79/F	1.0	Right breast, subareolar	Myxoid and chondroid	Excision
76	Ito et al. (2022) [47]	43/F	5.0	Left breast	Myxoid	Excision
77	Current case	66/F	5.5	Left breast	Chondroid	Mastectomy

Salivary gland analogous neoplasms of the breast range from benign pleomorphic adenomas to malignant adenoid cystic carcinomas. It has been suggested that pleomorphic adenoma probably starts as an intraductal papilloma [43]. Pleomorphic adenomas mostly occur in females; however, it has also been described in the male breast. The age of patients reported in the literature ranged from 19 to 85 years. The most common presentation was a palpable mass, and the most common location was retro-areolar. On physical examination, these neoplasms are difficult to differentiate from breast cancer, and no specific imaging features are described to help differentiate pleomorphic adenomas from malignant neoplasms. A core-needle biopsy might not be particularly helpful since limited sampling and the presence of any degree of atypia, architectural distortion or excessive myxoid or mesenchymal material can masquerade as a more ominous entity, leading to overdiagnosis of the lesion as malignant, including but not limited to a metaplastic breast carcinoma. Therefore, it is not uncommon that a correct diagnosis is made postoperatively on the surgical specimen after histopathological examination. The most common chromosomal rearrangements in pleomorphic adenomas of the salivary gland involve 8q12, containing the target gene PLAG1, or 12q13-15 with the target gene HMGA. The detection of PLAG1 and HMGAs translocations can be useful in confirming the rare diagnostically challenging pleomorphic adenomas in the salivary glands; however, the diagnostic utility of this technique in the breast is not certain [43].

Overall, clinicopathological and radiological correlation is of paramount importance when encountering a mixed neoplasm. The diagnosis of a benign lesion such as pleomorphic adenoma should also be considered in cases with well-circumscribed, bland ductal epithelium, and lack of significant atypia to avoid misdiagnosis and subsequent unnecessarily aggressive management for the patient.

Excision of the neoplasm with adequate margins is the treatment of choice in cases of pleomorphic adenoma of the breast. Although it is a benign neoplasm, an inadequate excision can result in recurrence and even multiple recurrences have been described. An excision of pleomorphic adenoma with clear margins should be ideally performed. Although there is no standardized margin distance, a margin as less as 3 mm has been described as adequate to prevent recurrence [48]. Any residual tumor may later manifest as recurrence and multiple recurrences are prone to malignant transformation.

## Conclusions

Pleomorphic adenoma is a benign neoplasm that most commonly occurs in the parotid gland but has only rarely been described in the breast. They are considered to be variants of intraductal papilloma or adenomyoepithelioma and are typically found in the subareolar region. The rarity of pleomorphic adenomas in the breast, as well as their unusual appearance, have contributed to overdiagnosis in this location. Pathologists should keep this tumor in mind whenever a tumor with a prominent chondromyxoid appearance is encountered, particularly in aspiration cytology or needle core biopsy material, despite the extreme rarity of this neoplasm in the breast.
